# Emotional face recognition when a colored mask is worn: a cross-sectional study

**DOI:** 10.1038/s41598-022-27049-2

**Published:** 2023-01-04

**Authors:** Sandrine Gil, Ludovic Le Bigot

**Affiliations:** Centre de Recherches sur la Cognition et l’Apprentissage (CeRCA UMR 7295), université de Poitiers, université de Tours, CNRS, Bât. A5 - MSHS, 5 rue Théodore Lefebvre, 86000 Poitiers, France

**Keywords:** Psychology, Human behaviour

## Abstract

Studies of the impact of face masks on emotional facial expression recognition are sparse in children. Moreover, to our knowledge no study has so far considered mask color (in adults and in children), even though this esthetic property is thought to have an impact on information processing. In order to explore these issues, the present study looked at whether first- and fifth-graders and young adults were influenced by the absence or presence (and color: pink, green, red, black, or white) of a face mask when asked to judge emotional facial expressions of fear, anger, sadness, or neutrality. Analysis of results suggested that the presence of a mask did affect the recognition of sad or fearful faces but did not influence significantly the perception of angry and neutral faces. Mask color slightly modulated the recognition of facial emotional expressions, without a systematic pattern that would allow a clear conclusion to be drawn. Moreover, none of these findings varied according to age group. The contribution of different facial areas to efficient emotion recognition is discussed with reference to methodological and theoretical considerations, and in the light of recent studies.

## Introduction

The COVID-19 pandemic meant that the wearing of face masks became mandatory or at the very least recommended. This unprecedented situation reopened the debate about the processing of information conveyed by the face. In particular, many recent studies have examined the extent to which a mask hiding the lower part of the face impacts the recognition of emotional facial expressions (EFEs). It is noteworthy that many people have tried to make the wearing of this health accessory less of a constraint by turning it into a fashion accessory, varying the color according to their mood and what they are wearing. However, color itself can carry an emotional meaning. Moreover, the vast majority of studies examining emotional expression recognition for masked faces have concerned adults. The objective of the present study was therefore to explore the effect of wearing a mask on EFE recognition from a developmental point of view (from child to adult) and to investigate whether the color of that mask modulates its effect.

Human faces convey rich emotional cues that are crucial for interpersonal communication, allowing partners to understand the emotional states of others or environmental information and adjust their behaviors accordingly^1–3^. Even though newborns demonstrate some remarkable early skills in dealing with EFEs (i.e. preference, discrimination)^4–6^, more sophisticated EFE recognition takes a lifetime to acquire. Moreover, studies suggest that the development of the ability to categorize EFEs differs according to the emotions, with some being fully recognized relatively late, such as surprise and disgust around the age of 8–10 years^7,8^.

A further, albeit controversial, difference in the way that adults and children process EFEs concerns the areas of interest. First, it remains unclear whether this processing is holistic or analytical. Holistic processing involves taking into account the relationship between the different key elements of the face to form a configural representation^9,10^. Analytical processing involves a specific area of the face (e.g. eyes vs. mouth) that is key to effective processing. The inverted face paradigm (i.e. testing whether inverted faces are less efficiently processed than upright ones) is deemed to prove the primacy of configural or holistic face processing in both adults^11^ and infants^12^, but results are less clearcut for children aged < 10 years^13^. Second, successful EFE recognition relies on specific diagnostic regions, depending on which emotion is being addressed. This perspective is derived from Ekman’s notion of emotional facial coding, involving a specific sets of action units (AUs) for each kind of emotion being expressed (Facial Action Coding System, FACS^14^). According to Ekman’s approach, which has been endorsed by numerous studies^15–18^, some parts of the face contain more useful information than others, depending on the emotion being conveyed: happiness and disgust are predominantly conveyed by the lower part of the face, and anger, fear and sadness by the upper part.

Given the above observations, it is not surprising that the systematic wearing of face masks at the height of the pandemic generated fresh interest in this subject. Results of recent studies conducted among adults generally indicate that when faces are masked versus unmasked, EFE recognition is less accurate (i.e. more confusion with other emotions) and emotional intensity is less well perceived^19–24^. However, these mask effects vary according to the emotion and may even be entirely absent. For example, Carbon (2020)^19^ found poorer performances in the masked condition for angry, disgusted, happy and sad faces, but not for fearful or neutral ones. In a study focusing on facial region predominance (upper vs. lower region), Langbehn et al. (2022)^24^ revealed that the perception of anger and surprise is less affected by the presence of a mask than that of happiness and disgust.

To our knowledge, the issue of whether mask wearing has an impact on children’s EFE recognition has so far only been addressed in two studies^25,26^. Ruba and Pollak (2020)^25^ asked children aged 7–13 years to perform a forced-choice task in which they were shown sad, angry or fearful facial expressions and had to choose between six options (sad, happy, angry, surprised, fearful, or disgusted). Faces were shown either uncovered, with sunglasses hiding the eyes, or with a surgical mask hiding the lower part of the face. Consistent with findings for adults, analyses showed that children’s emotion recognition was less accurate with masked faces than with uncovered faces, for all three tested emotions. The presence of sunglasses only affected angry and fearful faces, suggesting “that children inferred whether the face displayed sadness from mouth shape alone, whereas the information from the eye region was necessary for forming inferences about anger and fear” (^25^ p. 7). Carbon and Serrano (2021)^26^ also administered a forced-choice categorization task to children aged 9–11 years, with six different EFEs (angry, disgusted, fearful, happy, neutral and sad) and a choice between six different labels. The percentage of correct responses was high (89.9%) for uncovered faces, and lower for faces wearing a mask (77.7%). However, results revealed that this general detrimental impact of covered faces was very heterogeneous, with a substantial decrease in correct responses for disgust (uncovered faces: 85.1% vs. covered: 28.4%), only small decreases for fear (92.7% vs. 83.3%), happiness (98.8% vs. 93.4%) and sadness (82.7% vs. 72.5%), and unexpected increases for anger (89.3% vs. 93.6%) and neutral (90.9% vs. 95.2%).

Empirical evidence therefore confirms the detrimental effect of masks on EFE recognition, although this effect can be quite minor and vary according to the kind of emotion. However, some mask properties have so far been ignored. Masks can be of different styles and colors, such that they can become a kind of fashion accessory^27^. A large body of research indicates that color intrinsically carries emotional information and can therefore influence psychological functioning^28,29^. Specifically, red is more likely to prompt negative associations (i.e. aggressiveness, danger, failure)^30–32^, leading to avoidance behaviors^33,34^. By contrast, green is considered to prompt positive associations^35–37^. This implicit color-emotion association has already been tested in the context of EFE processing, with emotional faces being displayed either simultaneously with a color background^38–43^ or after a background used as a priming cue^43,44^ or even with the manipulation of the color of the face as such^45,46^. For example, Gil and Le Bigot (2014)^39^ found that the recognition of happy faces was enhanced when they were displayed simultaneously with a pink or green background, rather than with a gray one. By the same, token, these authors observed a more negative categorization of ambiguous emotional faces (i.e. surprised and neutral) when these were displayed against a red versus green or achromatic background^40^. Similarly, Young et al. (2013)^44^ found that the processing of angry faces was facilitated (i.e. faster categorization) when they were presented after a red background, rather than after a green or gray one. However, this effect did not extend to other negative facial expressions such as fear. This result highlights the importance of applying both dimensional (positive or negative valence)^47^ and discrete^48^ approaches to emotions. Finally, although achromatics are often used as control (i.e. neutral) colors, some studies have suggested that they can convey meaning, with black objects regarded as bad and white objects as good in Western society^49^. Interestingly, these implicit color-emotion associations and their impact have also been documented in school-aged children^50–53^. For instance, children choose black for *nasty* drawings^46^. In sum, the involvement of color in face processing can be considered in the context of the congruency effect: it has been well established that face processing is context-dependent^54–57^, and the target EFE may or may not be congruent with the contextual information. The face mask and its properties can be regarded as a contextual element for processing the upper half of the face.

Taken together, while some studies have explored the effect of wearing a mask on EFE recognition, this effect has been underinvestigated in childhood. Furthermore, it is unknown whether mask color modulates the effect according to the color-emotion association. To fill this gap, the present cross-sectional study was designed to investigate EFE recognition for unmasked faces versus faces masked with different colored masks, in both adults and school-aged children. According to the literature, one of the colors we used (i.e. red) conveys negative information. The same is true of the achromatic black, albeit to a lesser extent. Two other colors we used communicate positive information (green and pink), the same being true, though again to a lesser extent, of the achromatic white. We therefore expected colors with a negative valence to enhance the perception of facial negativity (congruent effect), and colors with a positive or neutral valence to reduce perceived negativity (incongruent effect). Moreover, based on the premise that some colors are associated with specific discrete emotions (e.g. red associated with anger), we expected (in)congruent effects to be observed selectively, and not for all negative emotional expressions.

## Method

### Participants

Participants were 82 children and adults. A total of four participants were excluded from the analyses, as two children did not follow the instructions correctly, and one adult and one child did not have normal color vision. All participants were screened for color vision with the short form of the Ishihara Color Vision Test^58^. The final sample included in the statistical analysis therefore contained 78 participants: 27 children (13 females) in first grade (*M* = 6.5, *SD* = 0.4), 27 children (14 females) in fifth grade (*M* = 10.6, *SD* = 0.5), and 24 adults (20 females) (*M* = 18.92, *SD* = 1.18). Adults were students who took part in exchange for course credits; they all gave their written informed consent. The children were recruited from French mainstream schools or leisure centers; all informed consent was obtained from their parents. The study was conducted in accordance with the principles expressed in the Declaration of Helsinki and was approved by the local ethics committee (CER-TP – Ethics Committee for the Research in humans of the universities of Tours and Poitiers).

### Material

**Figure 1:**
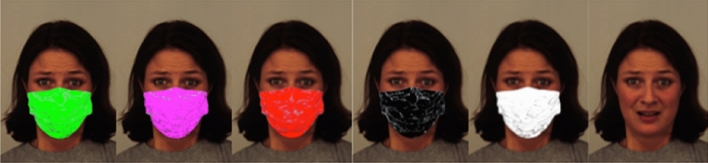
Example of a manipulated Karolinska emotional face displaying fear duplicated in the different mask conditions (green, pink, red, black, white and unmasked).

A PC controlled the experimental events using E-Prime 1.2 software (Psychology Tools, Pittsburg, PA). We extracted the faces of 16 persons (half women, half men) from the Karolinska Directed Emotional Faces^59^. Each person expressed three emotions (fear, anger, sadness) and neutrality. We choose these negative emotions because 1) according to Ruba and Pollak (2020)’s study, the upper region of the face is important to these emotional expressions, and 2) as already stated, they are assumed to be well recognized at a young age. Neutral faces were added as ambiguous stimuli^60,61^. Each stimulus was then duplicated with a surgical mask found in Google Images and declined in five different colors using Gimp software (green, pink, red, black, and white) for a total of 384 (16 × 4 × 6) unmasked and masked faces (see Fig. 1 for an example). Colors corresponded to the hue (H), saturation (S) and lightness (L) system, with hue variations solely for nonachromatics (green = 120° (H), 100% (S and L); pink = 300° (H), 100% (S and L); red = 0° (H), 100% (S and L); black = 0° (H), 0% (S and L); white = 0°(H), 0% (S), 100% (L)).

Participants responded on four 9-point Osgood-type scales (semantic differential scale)^62^ printed on laminated paper. More specifically, they were asked to express the perceived properties of each stimulus on semantic differential scales featuring two polarized options and a middle neutral alternative. This allowed us to the capture nuances in the ratings, as respondents assessed the stimuli in the context of the others. In order to apply both the discrete and dimensional approaches to emotion, three of the scales measured discrete emotions corresponding to the emotional faces used (anger vs. fear, anger vs. sadness, fear vs. sadness), and the fourth measured the valence dimension (negative vs. positive). The polarities of each scale were counterbalanced across participants, and were indicated by both words and black-and-white line drawings, to take account of the age of the younger children. Thus, fear was symbolized by a ghost, anger by a lightning bolt, sadness by rain, and positive and negative valences with a thumbs up or down.

### Procedure

The experimental session took place in a quiet room at the school or leisure center for the children, and in the laboratory for the adults, in 2021 when masks were mandatory in public areas. Participants were tested one at a time, seated in front of a computer screen. The experimenter began by explaining the general purpose of the task: to assess faces on the expression of different emotions. She gave a full description of all the scales, and took the time to give examples of how they worked. She made sure (particularly for the children) that the instructions were fully understood, and ended by stressing that there were no right or wrong answers, as she was interested in participants' personal opinion, and the aim was to respond fairly spontaneously.

The session was conducted in the presence of the experimenter, who controlled the presentation of the stimuli and noted the responses given orally by participants (whether child or adult). Participants had to assess each face on the four scales. Each trial began with a fixation cross, followed by the onset of the stimulus on the computer screen, which disappeared when the experimenter clicked on the mouse connected to the computer after the participant had responded. Each participant completed 48 trials (2 persons (1 male, 1 female) × 6 mask conditions × 4 facial emotions) pseudorandomly (same proportions of gender, mask condition, and emotion expressed) extracted from the total set of images.

## Results

### Data processing and analytical reasoning

The Osgood scale (i.e. semantic differential scale) is a bipolar scale with ordinal outcomes. Thus, for non-normal response distributions^63,64^, we performed a generalized linear mixed model (GLMM) for each dependent variable (i.e. each scale), using the SAS GLIMMIX procedure for categorical data (PROC GLIMMIX) in SAS version 9.4^65,66^. Among other things, these analyses make it possible to combine fixed effects (variables for which the levels included in the experiment represent all the levels of interest) with random effects, to account for potential variability across analysis units.

The multinomial models for ordinal outcomes used cumulative logit distribution (DIST = clogit). The cumulative logit-link function used proportional odds^67^. For all analyses, the initial model included all the independent variables and interactions as fixed factors, and participants and stimuli as a random factor: Mask condition (6, ref = Unmasked), EFE (4, ref = Neutral) and Age (3, ref = Adults) as fixed effects (note that references (ref) are included in brackets), and both participants and stimuli as random effects. The Satterthwaite correction^68^ was also applied, as the number of observations varied according to age group. If the statistical models failed to converge, the random effects that caused convergence issues were identified (automatically performed in SAS)^69^ and were removed without any impact on the analyses. The final random effects structure used in each model is specified below for each scale analysis.

If the general statistical model did not converge (the only case of Scale 2), the 9-point scale observations were converted on 3-point scale, with the values re-assigned as follows: 0, 1, 2 points were reassigned to 1; 3, 4, 5 were reassigned to 2; 6, 7, 8 were reassigned to 3. With the exception of this transformation, the reasoning and statistical analyses were similar for all scales.

When a significant effect was obtained, least square means were estimated using the LSMEANS function of SAS. Least square means are not estimates of event probabilities, but rather estimates of the linear predictors on the logit scale, and are therefore estimated log odds^66^. In order to obtain event probabilities, we therefore needed to apply the inverse-link transformation by specifying the ILINK option in the LSMEANS statement of SAS. As multinomial mixed models in SAS GLIMMIX PROC do not allow least square means to be directly reported, we used PROC PLM to restore the models^70^.

To explore the significant interactions, we used the SLICE option (simple effect analysis)^66,71^. This involves testing the effect of a given factor at the different levels of the other factors^72^. Finally, all reported *p* values were adequately adjusted for multiple comparisons, with the Bonferroni adjustment and ADJDFE = ROW setting^66^.

As the polarities of each 9-point scale (or 3-point scale for Scale 2) were counterbalanced across participants for methodological reasons, the dataset was unified prior to statistical processing. Scale 1 referred to anger (0) versus fear (8), Scale 2 to anger (0→1) versus sadness (8→3), Scale 3 to sadness (0) versus fear (8), and Scale 4 to negative (0) versus positive (8).

### Scale 1 (anger vs. fear)

**Figure 2:**
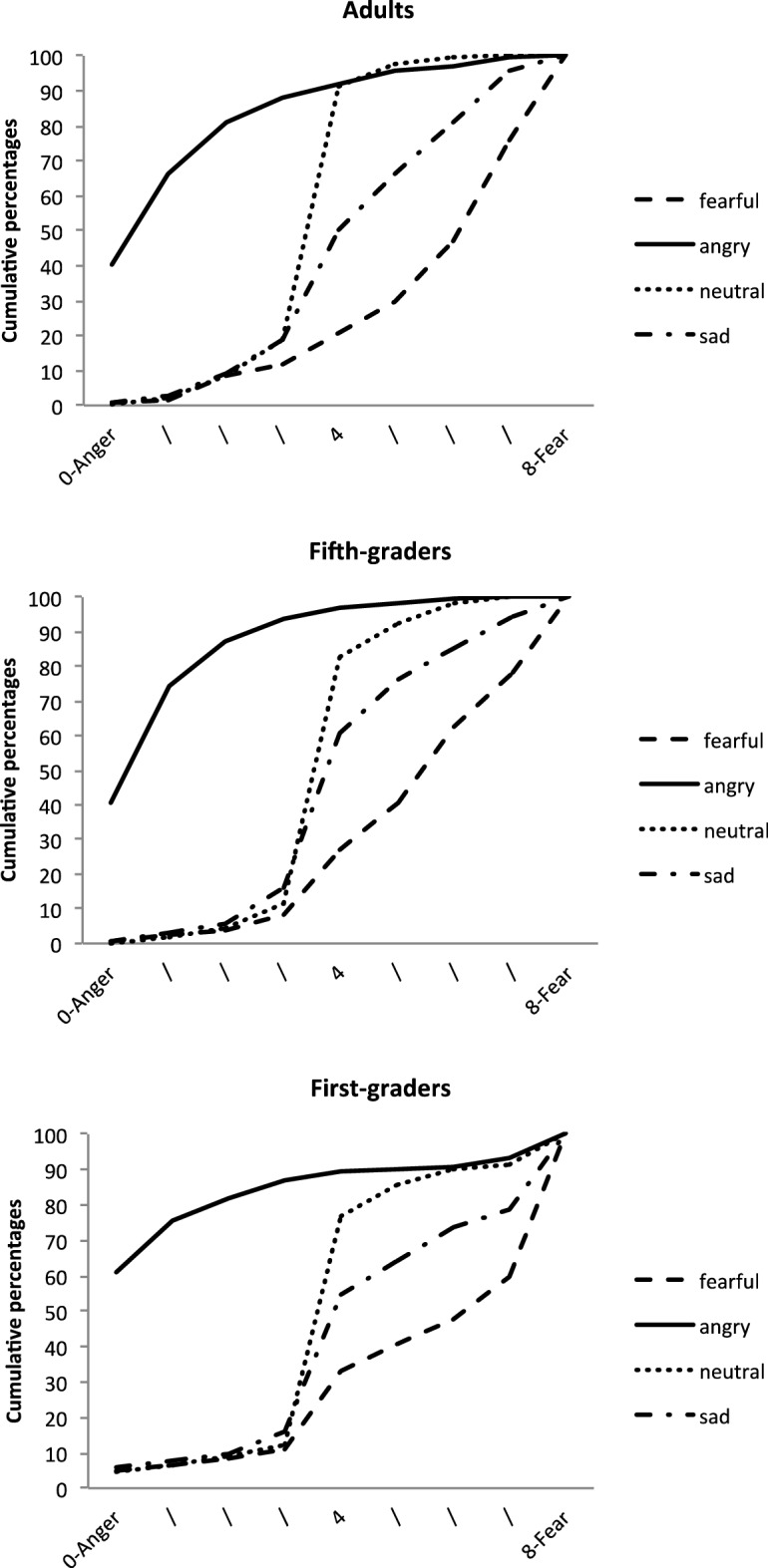
Cumulative percentages of the perception of EFE on Scale 1, for each age group.

The final model included all the independent variables and interactions as fixed factors, and participants as a random factor. It yielded a significant main effect of EFE, *F*(3, 3665) = 703.49, *p* < 0.0001, and significant interactions between EFE and Age, *F*(6, 3665) = 6.76, *p* < 0.0001, and between EFE and Mask condition, *F*(15, 3665) = 1.88, *p* = 0.021. None of the other main or interaction effects were significant. As illustrated in Fig. 2, the main effect of EFE corresponded to generally good EFE recognition, with angry faces being rated as conveying more anger, and fearful faces as expressing more fear. Neutral and sad faces were adequately rated in the middle of this scale. All pairwise comparisons between EFEs were significant (all *p*s < 0.001).

The EFE x Age interaction showed that ratings for some emotions varied across age. More specifically, there were no simple effects of age for fearful (*F*(2, 216.8) = 2.10, *p* = 0.12) and sad (*F*(2, 224.9) = 2.68, *p* = 0.07) faces. By contrast, age had significant effects on the perception of angry, *F*(2, 242) = 4.77, *p* = 0.009, and neutral, *F*(2, 232.7) = 3.94, *p* = 0.021, faces, with ratings differing significantly between first graders and adults (*t*(246.5) = 3.04, *p* = 0.008, and *t*(232.8) = − 2.79, *p* = 0.017).

**Figure 3:**
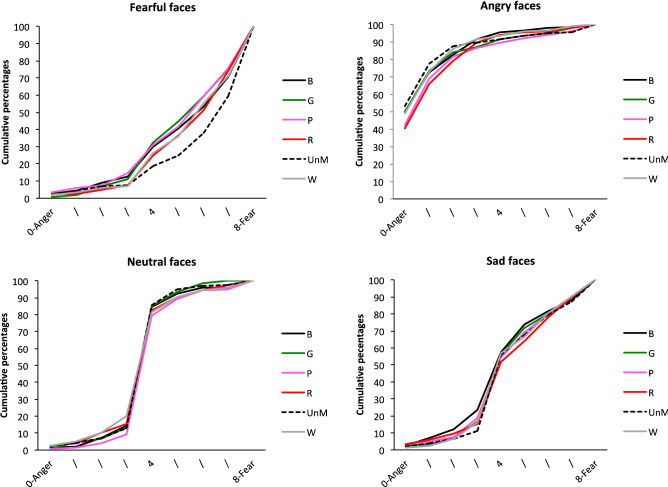
Cumulative percentages of the perception of each EFE for Scale 1, as a function of mask condition (faces wearing a *B* black, *G* green, *P* pink, *R* red, *W* white, *mask*; *UnM* unmasked face).

The EFE x Mask condition interaction indicated that the emotional assessment depends on mask condition. In sum and as illustrated in Fig. 3, whereas ratings were similar for angry, sad and neutral faces whatever the mask condition (all *p*s > 0.1), this was not the case for fearful facial expressions, for which we observed an effect of mask condition, *F*(5, 3665) = 4.12, *p* = 0.001. Fearful facial expressions were more likely to be judged to be expressing fear in the unmasked condition than in some of the colored mask conditions: faces wearing a pink (*t*(3665) = 3.90, *p* = 0.001), green (*t*(3665) = 3.93, *p* = 0.001) or black (*t*(3665) = 3, *p* = 0.04) mask. The other mask conditions were not significant.

### Scale 2 (anger vs. sadness)

As mentioned earlier, as no statistical model converged for this scale, the analyses reported below were performed on a 3-point scale. The final model included all the independent variables and interactions as fixed factors, and participants and stimuli as random factors. As in Scale 1, the main effect of EFE was significant, *F*(3, 3671) = 454.11, *p* < 0.0001, with good recognition of each EFE: angry expressions were rated as conveying more anger, and sad expressions as conveying more sadness, while neutral and fearful faces were rated indifferently (for all multiple comparisons, *p*s < 0.0001). The statistical model also revealed an EFE x Age interaction, *F*(6, 3671) = 5.96, *p* < 0.0001, and an EFE x Mask condition interaction, *F*(15, 3671) = 2.59, *p* < 0.001. No other main or interaction effect was significant.

**Figure 4:**
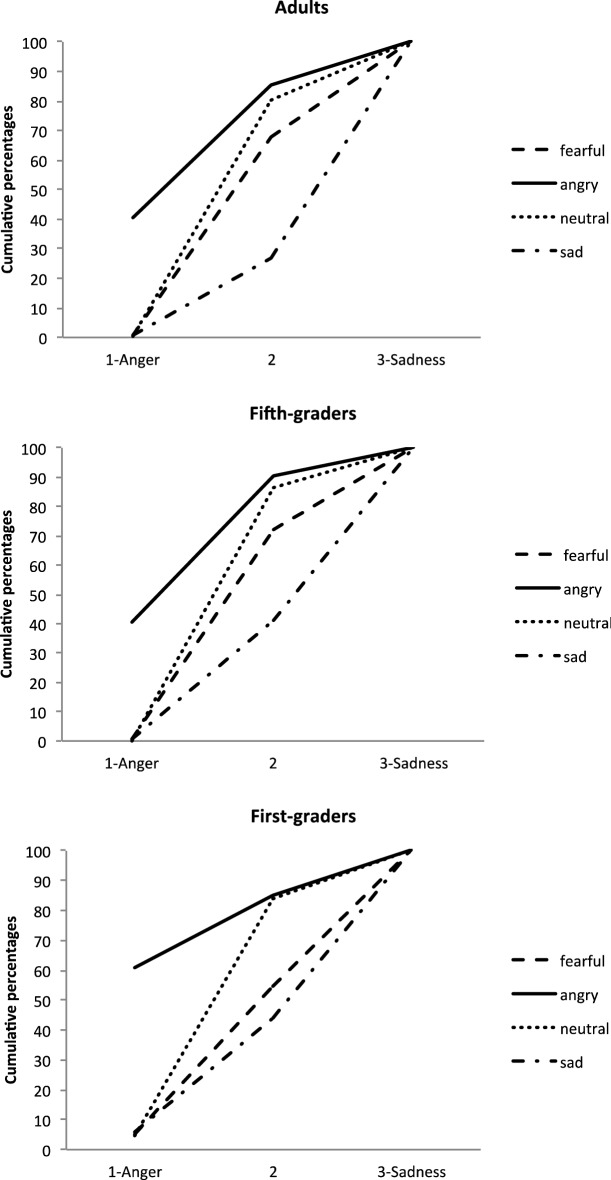
Cumulative percentages of the perception of EFE on Scale 2, for each age group.

A suggested in Fig. 4, examination of the simple effects for the EFE x Age interaction showed that there was no effect of age group for angry (*F*(2, 252.1) = *p* = 0.70) and neutral (*F*(2, 244.8) = 7.66, *p* = 0.68) faces. By contrast, for sad faces, *F*(2, 244.8) = 7.66, *p* = 0.0006, adults’ ratings differed from those of both first graders (*t*(274,8) = 2.94, *p* = 0.0005) and fifth graders (*t*(279.4) = 2.94, *p* = 0.011). Differences between groups also emerged for fearful faces, *F*(2, 188) = 3.52, *p* = 0.032, but less strongly, with first graders’ ratings being only marginally different from those of fifth graders (*t*(188.7) = − 2.23, *p* = 0.08,) and adults (*t*(188.9) = − 2.35, *p* = 0.06).

**Figure 5:**
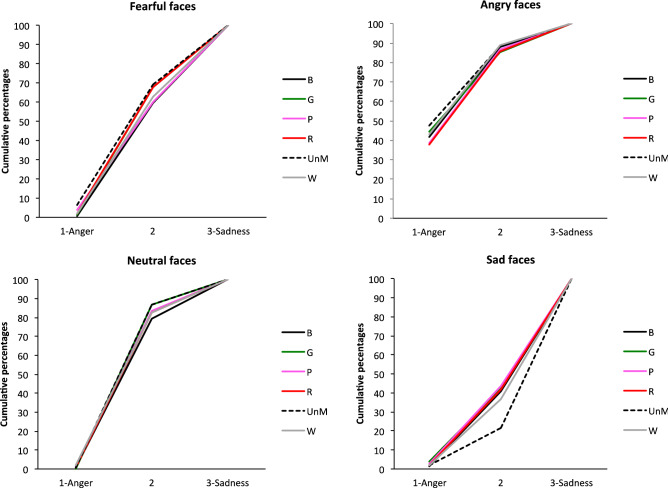
Cumulative percentages of the perception of each EFE for Scale 2, as a function of mask condition (faces wearing a *B* black, *G* green, *P* pink, *R* red, *W* white, *mask; UnM* unmasked face).

As illustrated in Fig. 5, the EFE x Mask condition interaction stemmed from the fact that ratings differed according to mask condition for faces expressing sadness, *F*(5, 3671) = 4.62, *p* = 0.0003. In other words, for sad facial expressions, data revealed a mask condition effect, with faces expressing sadness rated as sadder in the unmasked condition than in all the masked conditions: black (*t*(3671) = 4.02, *p* < 0.0001); green (*t*(3671) = 4.04), *p* < 0.0001), pink (*t*((3671) = 4.41), red (*t*(3671) = 3.95, *p* < 0.0001) and white (*t*(3671) = 3.13, *p* = 0.0017). No other significant effect emerged for the other EFEs, with the exception of neutral faces, *F*(5, 3671) = 2.28, *p* = 0.044, for which there was only a marginal simple effect that does not warrant comment under the parsimony principle.

### Scale 3 (sadness vs. fear)

The final model included all the independent variables and interactions as fixed factors, and participants as a random factor. Analysis revealed significant main effects of EFE, *F*(3, 3665) = 220.35, *p* < 0.0001, suggesting that EFE recognition depended on the scales’ polarities (all *ps* < . 0001, except–logically–for the difference between neutral and angry faces, which were given a middle neutral rating (*p* = 1)). Analysis also revealed a main effect of Age, *F*(2, 73.74) = 8.05, *p* = 0.0007, as ratings differed between first graders and both adults, *t*(74.01) = − 3.92, *p* = 0.0006, and fifth graders, *t*(73.76) = − 2.63, *p* = 0.031. These main effects were modulated first by an EFE x Age interaction, *F*(6, 3665) = 9.76, *p* < 0.0001, and second by an EFE x Mask condition interaction, *F*(15, 3665) = 2.20, *p* = 0.005.

**Figure 6:**
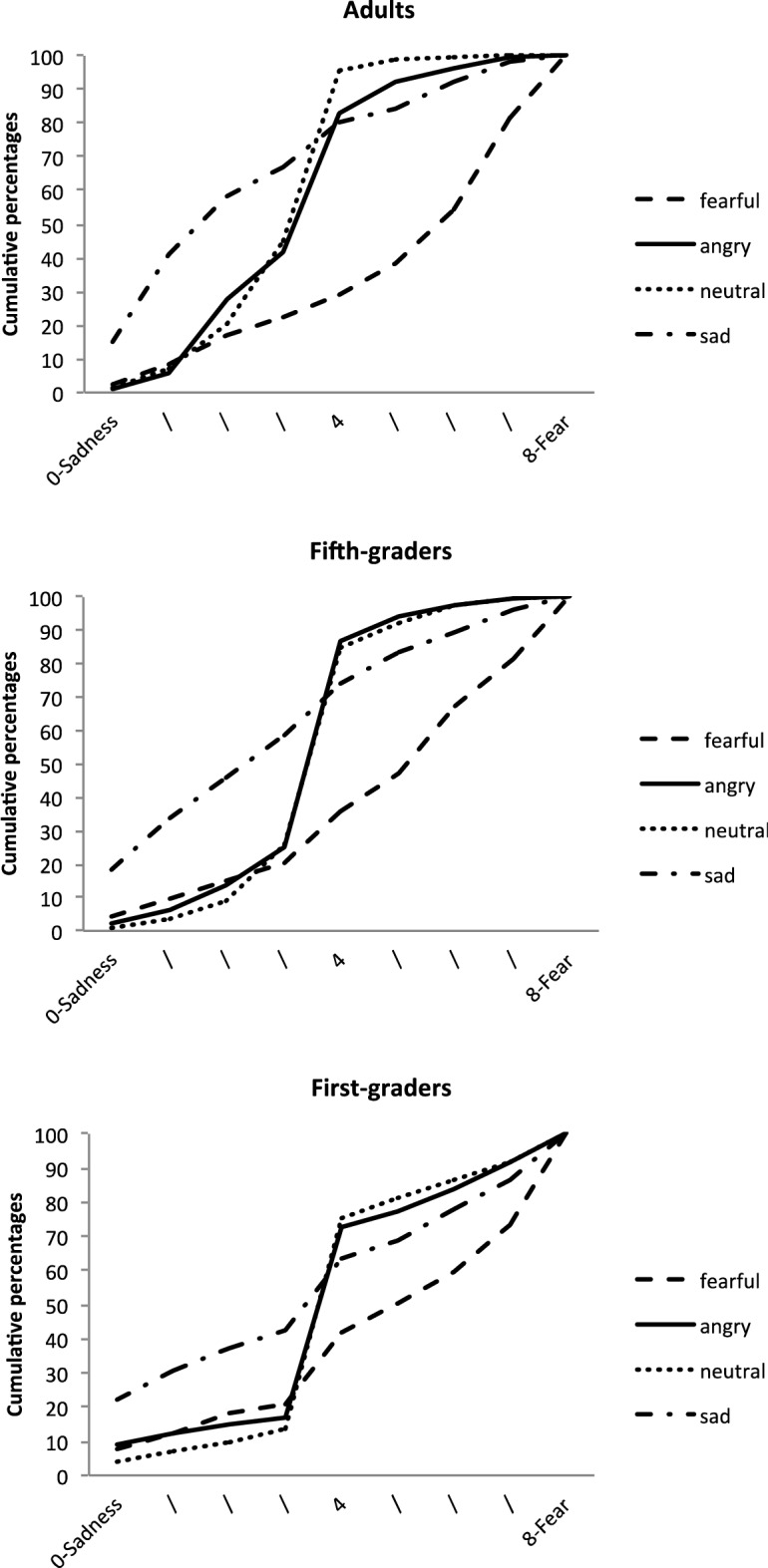
Cumulative percentages of the perception of EFE on Scale 3, for each age group.

**Figure 7:**
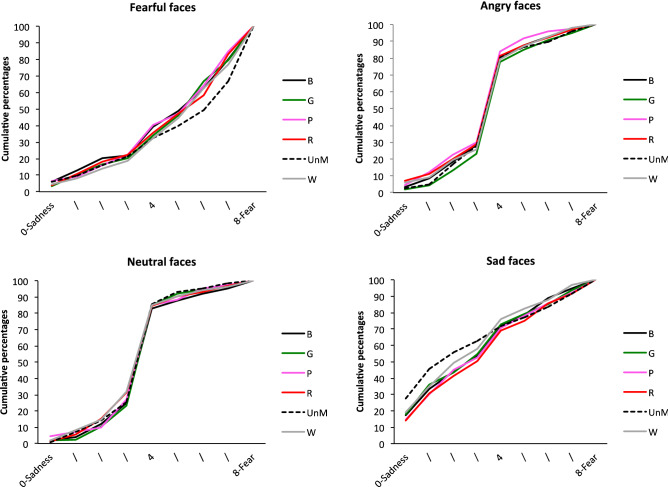
Cumulative percentages of the perception of each EFE for Scale 3, as a function of mask condition (faces wearing a *B* black, *G* green, *P* pink, *R* red, *W* white, *mask*; *UnM* unmasked face).

As illustrated in Fig. 6, the EFE x Age interaction reflected the fact that there was no effect of age for fearful faces (*p* = 0.27), but a significant effect of age for the other EFEs: angry, *F*(2, 185.3) = 7.09, *p* = 0.0011; sad, *F*(2, 183.5) = 15.55, *p* < 0.0001; and neutral, *F*(2, 185.4) = 10.69, *p* < 0.0001. More specifically, for angry faces, ratings differed between adults and first graders (*t*(185.8) = − 3.67, *p* = 0.0009)*.* For sad faces, first graders’ ratings differed from both fifth graders’ (*t*(184.3) = − 3.55, *p* = 0.0015), and adults’ (*t*(184.8) = − 5.49, *p* < 0.0001) ratings. For neutral faces, adults’ ratings differed from those of both first graders (*t*(186.1) = − 4.62, *p* < 0.0001) and fifth graders (*t*(184.9) = − 2.56, p = 0.034). The EFE x Mask interaction showed that there was no mask effect for either angry (*F*(5, 3665) = 0.78, *p* = 0.56) or neutral (*F*(5, 3665) = 0.24, *p* = 0.94) faces, but there were effects for fearful, *F*(5, 3665) = 2.70, *p* = 0.02, and sad, *F*(5, 3665) = 3.26, *p* = 0.006, ones (see Fig. 7). For fearful faces, simple effects revealed a significant difference between unmasked faces and both black (*t*(3665) = 2.99, *p* = 0.042) and pink (*t*(3665) = 3.20, *p* = 0.021) mask conditions. For sad faces, ratings differed between the unmasked condition and the pink (*t*(3665) = − 3.19, *p* = 0.022) and red (*t*(3665) = − 3.45, *p* = 0.009) mask conditions.

### Scale 4 (negative vs. positive)

**Figure 8:**
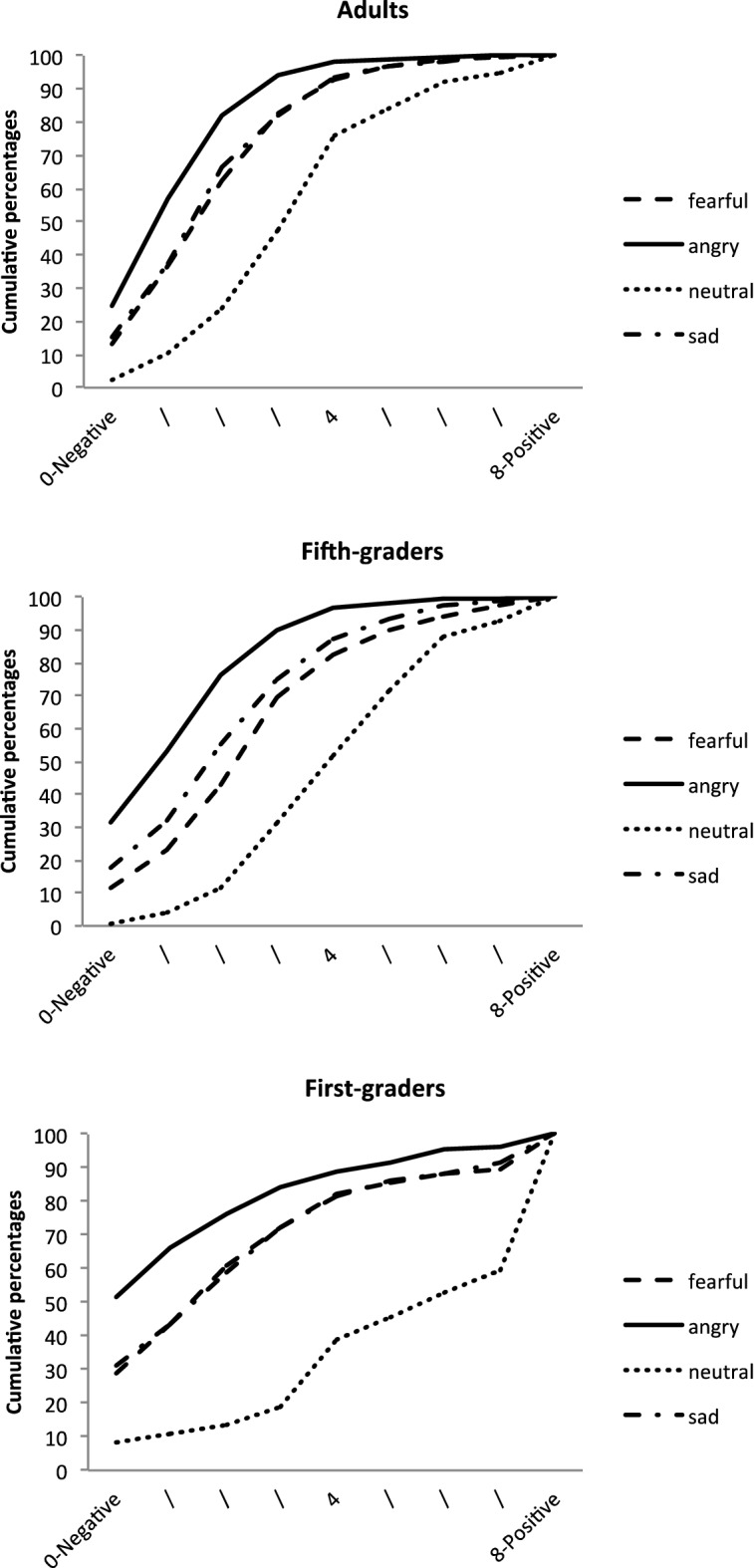
Cumulative percentages of the perception of EFE on Scale 4, for each age group.

The final model included all the independent variables and interactions as fixed factors, and participants and stimuli as random factors. The model revealed a main effect of EFE, *F*(5, 3665) = 4.90, *p* = 0.0002. This suggested good recognition of emotions, with angry faces assessed as more negative, and neutral faces as less negative. All these pairwise comparisons were significant (all *ps* < 0.0001), except for sad versus fearful faces which were judged as similarly negative, *t*(3665) = − 2.12, *p* = 0.20. This main effect was modulated by a significant EFE x Age interaction, *F*(6, 3665) = 31.56, *p* < 0.0001. Examination of simple effects showed no effects of age for the different EFEs, except for neutral faces, *F*(2, 87.21) = 14.09, *p* < 0.0001, where all groups differed from each other: first graders vs. fifth graders (*t*(87.72) = − 3.28, *p* = 0.0015); first graders vs. adults (*t*(88.52) = − 5.25, *p* < 0.0001); and fifth graders vs. adults (*t*(85.47) = −  2.09, *p* = 0.039) (see Fig. 8).

**Figure 9:**
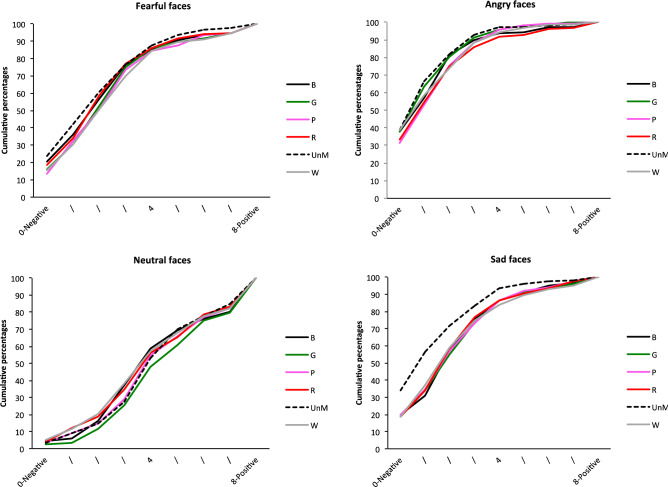
Cumulative percentages of the perception of each EFE for Scale 4, as a function of mask condition (faces wearing a *B* black, *G* green, *P* pink, *R* red, *W* white, *mask*; *UnM* unmasked face).

The analysis also yielded a main effect of Mask condition, *F*(5, 3665) = 407.34, *p* < 0.0001, with unmasked faces generally rated as more negative, compared with all the masked faces: green, *t*(3665) = − 4.27, *p* < 0.001; pink, *t*(3665) = − 4.01, *p* < 0.001; red, *t*(3665) = − 3.06, *p* = 0.033; white, *t*(3665) = − 3.80, *p* < 0.01; and marginally black, *t*(3665) = − 2.77, *p* = 0.08. This effect was modulated by a significant EFE x Mask condition interaction, *F*(15, 3365) = 1.82, *p* = 0.026. Analysis of simple effects revealed that there was no mask effect for emotional faces (all *ps* > 0.1), except for sad faces (see Fig. 9). For the latter, unmasked faces were rated as more negative than all the masked faces: green, *t*(3665) = − 4.38, *p* < 0.001; pink, *t*(3665) = − 4.14, *p* < 0.001; red, *t*(3665) = − 3.98, *p* = 0.001; white, *t*(3665) =  − 4.07, *p* < 0.001; and black, *t*(3665) = − 4.33, *p* < 0.001.

## Discussion

The 2020–2021 pandemic prompted users and, obviously, scientists, to think about the impact of wearing a face mask. The present study tackled a fundamental but very apposite question about whether the presence of a mask affects EFE processing. Our aim was to add to recent data on the issue by conducting a study among children, who have so far been largely ignored by researchers^25,26^, and to raise an additional and original question of interest about face-mask color, insofar as color is an element of information processing. To this end, adult and child (first- and fifth-graders) participants performed an EFE recognition task in a physical laboratory setting (which can be considered a strength, given that studies have mostly featured online assessments to date), and we manipulated the emotions expressed by the faces and the mask condition (i.e. unmasked vs. masked with different colors).

Regarding the developmental aspect of the study, results yielded encouraging results. Unlike age-related results appeared, EFE ratings differed according to scale, but there was no significant interaction with mask condition, suggesting that the manipulation of the stimuli (unmasked vs. masked with different colors) did not have a significant age-related impact. This is consistent with some previous results showing good recognition for masked faces in children^25,26^, albeit with poorer performances for some specific EFEs. Nevertheless, cross-sectional studies of EFE recognition for masked faces among children are sparse, as the present study was the only one to our knowledge to test different age groups. Ruba and Pollak (2020)’s study only included children^25^, but the results that Carbon and Serrano (2021)^26^ obtained in children can be indirectly compared with the results for adults in Carbon (2020)^19^. It therefore seems reasonable to state at the present time that mask-wearing has a relatively limited impact on EFE recognition across all age groups, as already underlined by some authors^25,73^. It is also worth noting that when analyses yielded an age effect, fifth-graders produced intermediate ratings between adults and younger children, strengthening existing data on sociocognitive development^7,8^. Other studies dealing with the wearing of face masks, but investigating identity recognition, have yielded mixed results, with a few cross-sectional studies involving children. Some revealed an inversion effect in both adults and children, with a more pronounced effect in children^74^ whereas others failed to find an inversion effect in adults^21^.

It is important to point out that the results have to be considered in relation to the methodology used, and it can sometimes be complex to compare them, as different methodologies engage different mechanisms (for a similar discussion, see^22^). For instance, the majority of published studies used a forced-choice paradigm with more or fewer options for the answers (i.e. either the same number of options as emotions to be processed, or more). In addition, there was no “Don’t know” option. In the present study, participants were asked to rate the stimuli on semantic differential scales between two polarized options. Thus, whereas forced-choice paradigms allow measures to be based on some specific labels, they have the disadvantage of potentially eliciting artificially high performances, as participants can respond through a process of elimination^75^, such that the lower the number of options and the simpler the task, the higher the score. By contrast, the use of differential scales by definition enables participants to provide ratings with more or less each of the proposed options and to express *neither*. However, this leads to less straightforward categorization.

Regarding the effect of mask, two EFEs (i.e. sadness and fear) in this study seemed to have a very specific pattern of results. First, analysis of ratings on Scales 2, 3 and 4 revealed that faces expressing sadness were perceived of more as expressing sadness (Scales 2 and 3) or as being more negative (Scale 4) in the unmasked condition than in the pink and red mask conditions for Scale 3, and whatever the color of the mask for Scales 2 and 4. This particular sensitivity of sadness recognition to the presence of a mask is congruent with some previous studies suggesting a particular decrease in sad face recognition with a mask^19,20,22,26^. More specifically, after disgust (not used in the present study), sadness appears to have been the expression most impacted in several studies performed in adults^20,22^. However, this result is to be contrasted with those reported by Ruba and Pollak (2020)^25^ or obtained before the mask issue arose^64^, which suggest that sad expressions are better processed by focusing on the upper part of the face. In fact, these contrasting results are not irreconcilable and can be linked to the two main AUs known to be involved in the expression of sadness: one in the top half of the face (AU1: inner brow raiser) and one in the bottom half (AU 15: lip corner depressor)^14^.

Second, results for Scales 1 and 3 revealed a stronger categorization of fearful faces when unmasked, compared with some colored masked conditions. This indicates that the processing of fearful expressions is sensitive to mask wearing, as shown in some studies^20,22,26^ but not others^19,25^. Once again, these results may be related to the characteristic pattern of fear expression, involving both upper and lower face AUs. However, an alternative explanation is the strong confusion reported in the literature between fear and surprise face patterns in both adults and children^76–79^, as these emotions share two characteristic upper-face AUs (AU2: Outer brow raiser; AU5: upper lid raiser). Although surprise was not a response option in any of the bipolar scales, participants may have thought about this possibility, thus muddling their perception of the fearful expressions. Furthermore, Scale 1 presented anger and fear as polar opposites. These are the only primary emotions to share two upper-face AUs (AU4: brow lowerer; AU5: upper lid raiser). For this bipolar scale, fearful and angry faces with masks could therefore be particularly ambiguous, in contrast to unmasked faces. However, although this is an interesting explanation, it is not corroborated by the results for angry faces, for which the effect of mask failed to reach significance.

Our data do not support an effect of mask wearing on the processing of angry and neutral expressions. Although a non-result should be viewed with caution, it is worth noting that this is consistent with some previous studies. For example, a number of studies have suggested that the upper part of the face is important for anger recognition both by adults^24^ and by children^25^, and even enhances children’s processing of masked faces expressing anger and neutrality^26^. Taken together, the results of both the present study and previous ones suggest that processing of the eye region is an efficient means of recognizing anger and neutrality.

Finally, regarding the color issue, results did not reveal a systematic pattern that would allow a clear conclusion to be drawn. It is worth noting that whereas Scales 2 and 4 showed a general difference between the faces without a mask and the set of masked faces, whatever the color (for sad faces), Scales 1 and 3 only showed differences for some of the colored masks. The pink mask appeared in the results of all four scales. Its systematic influence can be viewed as consistent with the color literature, as pink^39,80^ has positive associations, even if it has been researched less than other colors. In other words, fearful or sad expressions were perceived of as evoking the target emotion more when the faces were unmasked than when the faces wore a mask of a positive color like pink. However, this interpretation should be viewed with caution, for generally speaking, it seems that it was the wearing of the mask per se, and not the different colors, that accounted for our results.

To conclude, the present study provided a further analysis of adults’ and children’s processing of masked EFEs, including an examination of mask color. Findings are consistent with the view that wearing a mask does not dramatically affect the ability to process facial information in either population. Moreover, consistent with previous studies, the presence of a mask seemed to have a heterogeneous impact depending on the nature of the emotion, affecting only fear and sadness recognition. Finally, the lack of consistency between some previous studies may be accounted for by methodological factors, but the present study also had several limitations. For instance, whereas we used static stimuli, it is better to explore the processing of dynamic rather than static facial expressions, as the former are more ecological (for a large cross-sectional investigation, see^81^). However, recent studies using dynamic EFEs yielded a similar conclusion, with more confusion with masked versus unmasked faces as a function of EFE, but generally good performances^23,24^. Concerning colored patterns and designs, some might point out that in real life, colored masks do not necessarily correspond to the pure hues we used here for methodological reasons. In addition, we did not modify the brightness of the EFE stimuli, even though the colors used for the masks were very bright. This contrast may have influenced the perceptual processing of each part of the face. Moreover, we did not use a spectrophotometer to measure the properties of the colors as they were actually displayed on the computer monitor, and virtually none of the experimental sessions took place in a laboratory setting, where conditions could have been more carefully controlled. Another potential limitation regarding color is that its effect is context dependent, as emphasized by color-in-context theory^28,29^. Some studies exploring mask perception have shown that, contrary to their classically negative perception, masks can communicate positive representations like trustworthiness and security during a pandemic^82,83^. The manipulation of a positive versus negative interactional context might therefore modulate the meaning of the mask, and thus shed further light on the effect of colored masks in face processing.

## Data Availability

The datasets analysed during the current study are available from the corresponding author on reasonable request.
